
JOURNAL INFORMATION
==============================
NLM Title Abbreviation: Vet Rec Open
Journal ID: Vet Rec Open
NLM Journal ID: 101653671
Journal ID: VRO2

Veterinary Record Open

EISSN: 2052-6113
Publisher: Wiley

ARTICLE INFORMATION
==============================
PMCID: PMC8110111
DOI: 10.1002/vro2.11
Article ID: VRO211
Article version: 1
Subjects: Issue Information, Issue Information

Issue Information

Electronic publication date: 2021 Mar 30
Publication date: 2021 Dec
Volume: 8
Issue: 1
Electronic Location ID: e11
Copyright: © 2021 The Authors. Veterinary Record Open published by John Wiley & Sons Ltd on behalf of British Veterinary Association.
License: This is an open access article under the terms of the http://creativecommons.org/licenses/by/4.0/ License, which permits use, distribution and reproduction in any medium, provided the original work is properly cited.
License URL: https://creativecommons.org/licenses/by/4.0/

Figure count: 0
Table count: 0
Page count: 2

source-schema-version-number: 2.0
cover-date: December 2021
details-of-publishers-convertor: Converter:WILEY_ML3GV2_TO_JATSPMC version:6.0.2 mode:remove_FC converted:10.05.2021

==============================
ISSN 2399-2050

### Vet Record Open

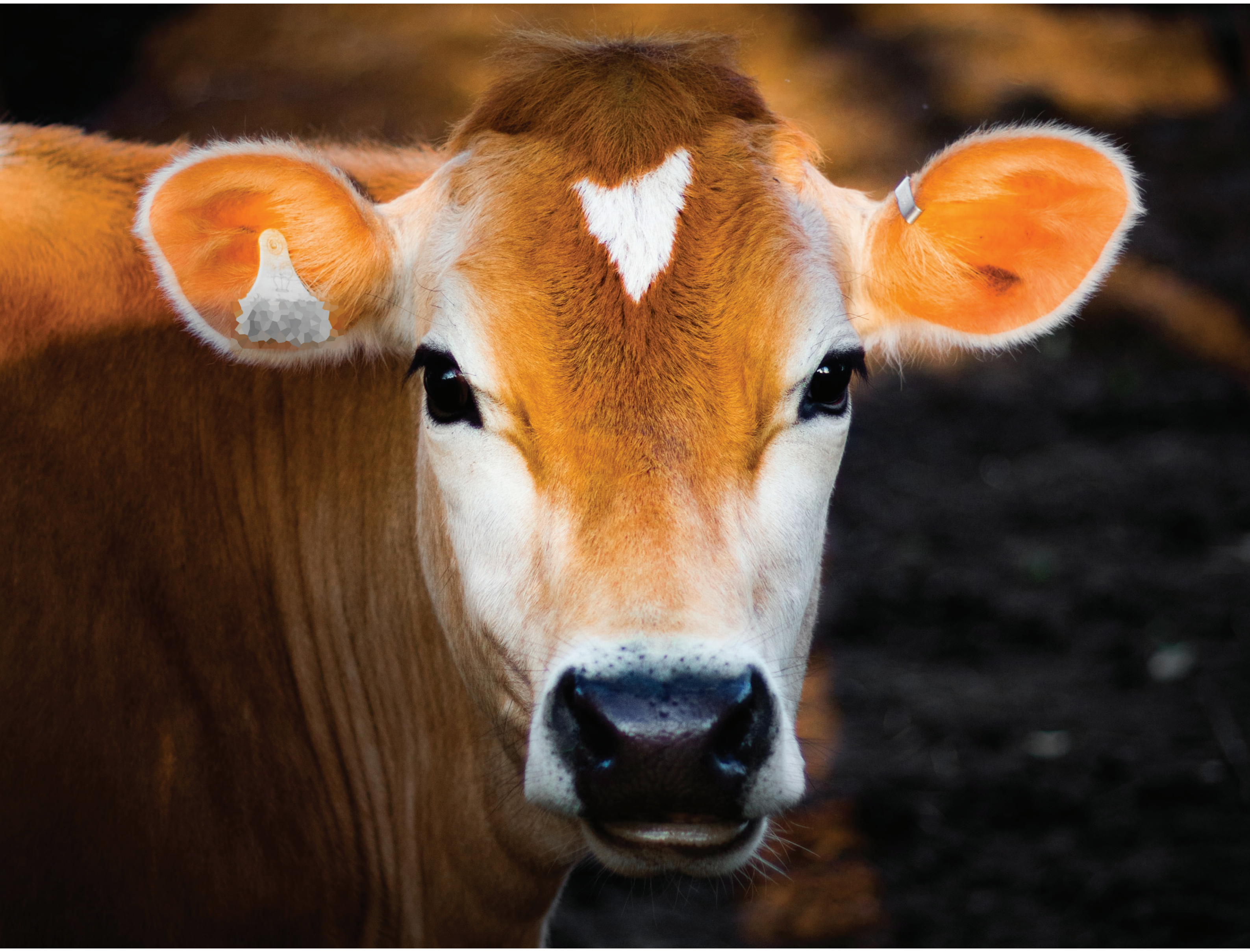

BVA British Veterinary Association

WILEY

### Vet Record Open

**Chief Veterinary Adviser**

**Professor The Lord Trees, MRCVS, FMedSci, HonFRSE**
*House of Lords, London, UK*
***Area of expertise:*** Veterinary parasitology, Veterinary policy, Neglected tropical diseases
orcid.org/0000-0001-8509-4728

**Editor**

**Dr Aiden Foster PhD, DipACVD, FHEA, MRCVS**
*University of Bristol, Bristol, UK*
***Area of expertise:*** *Dermatology, Pathology*
orcid.org/0000-0003-3808-8434

**Editorial Board**

**Dr Priya Bapodra, BVetMed, MSc, Dipl ACZM, MRCVS**
*Columbus Zoo and Aquarium, Ohio, USA*
***Area of expertise:*** *Zoo medicine, Hoofstock and megavertebrate medical management*
orcid.org/0000-0003-1433-9577

**Dr Elena Barrio, MVDr, PhD, MRCVS**
*The Donkey Sanctuary, Sidmouth, UK*
***Area of expertise:*** *Equine (specifically donkey) preventive medicine*
orcid.org/0000-0002-5543-3351

**Dr Jacqueline M Cardwell, MA, VetMB, PhD, MSc, FHEA, MRCVS**
*Royal Veterinary College, London, UK*
***Area of expertise:*** *Equine asthma, Biosecurity, Infectious diseases*
orcid.org/0000-0002-9065-3253

**Dr James Carmalt, MA, VetMB, MVetSc, PhD, FRCVS, DABVP, DAVDC, DACVSMR, DACVS**
*Western College of Veterinary Medicine, Saskatchewan, Canada*
***Area of expertise:*** *Equine surgery*

**Dr Tom Cave, PhD**
*Cave Veterinary Specialists, West Buckland, UK*
***Area of expertise:*** *Haemotology, Oncology, Cats, Dogs, Endoscopy*

**Prof Beniamino T Cenci-Goga, DVM, PhD**
*University of Perugia, Perugia, Italy*
***Area of expertise:*** *Food safety, Zoonoses, Public health, Epidemiology, Foodborne diseases*
orcid.org/0000-0002-3993-6258

**Dr Matthieu Cousty, PhD**
*Centre Hospitalier Vétérinaire Equin de Livet, St Michel de Livet, France*
***Area of expertise:*** *Equine surgery*

**Dr Fátima Cruz-López, MRCVS, FMedSci, HonFRSE**
*VISAVET Health Surveillance Centre, Madrid, Spain*
***Area of expertise:*** *Equine health surveillance*
orcid.org/0000-0002-1142-3944

**Dr Luisa De Risio, DVM, PhD, DECVN, FRCVS, RCVS, EBVS**
*Linnaeus Group, Shirley, UK*
***Area of expertise:*** *Veterinary neurology*
orcid.org/0000-0001-9005-4165

**Dr Franck Forterre, ECVS**
*Vetsuisse Faculty of Bern, Bern, Switzerland*
***Area of expertise:*** *Neurosurgery, Intervertebral disc disease, Atlantoaxial instability*
orcid.org/0000-0001-9026-9326

**Dr Allison German, BVSc, MSc, PhD, MRCVS**
*Independent Veterinary Consultant; Honorary Research Fellow at University of Liverpool, Wirral, UK*
***Area of expertise:*** *Feline and canine internal medicine, Virology, Infectious disease*
orcid.org/0000-0002-1846-751X

**Dr Rhonda M Hoffman, PhD, PAS, Dipl ACAN**
*Middle Tennessee State University, Tennessee, USA*
***Area of expertise:*** *Equine nutrition, Equine metabolic syndrome, Minimal model of glucose kinetics*
orcid.org/0000-0001-5879-4390

**Dr Aarti Kathrani, BVetMed, PhD, DACVIM, DACVN, FHEA, MRCVS**
*Royal Veterinary College, Hatfield, UK*
***Area of expertise:*** *Small animal nutrition and gastroenterology*
orcid.org/0000-0001-5569-794X

**Dr Adriaan Kitshoff, PhD**
*University of Pretoria, Onderstepoort, South Africa*
***Area of expertise:*** *Small animal surgery*

**Dr Miguel A Moreno, PhD**
*Complutense University of Madrid, Madrid, Spain*
***Area of expertise:*** *Antimicrobial resistance and use, Veterinary epidemiology and microbiology*
orcid.org/0000-0002-5357-3684

**Dr Lisa Morrow, DipAppChemBiol, BMLSc, DVM, MSc, DLSHTM, MRCVS**
*University of Nottingham, Sutton Bonington, UK*
***Area of expertise:*** *Epidemiology*
orcid.org/0000-0003-3001-1088

**Dr Eugenia Natoli, PhD**
*Interzonal Dog Shelter, Rome, Italy*
***Area of expertise:*** *Social behaviour and reproductive strategies in mammals, Animal welfare*
orcid.org/0000-0002-9232-7048

**Mr C Daniel Parker, BA, VetMB, CertPMP, DipECPVS, FRCVS**
*Slate Hall Veterinary Practice, Metheringham, UK*
***Area of expertise:*** *Poultry veterinary medicine and production*
orcid.org/0000-0002-0066-4902

**Dr Adolfo Paz Silva, PhD**
*University of Santiago de Compostela, Santiago, Spain*
***Area of expertise:*** *Cattle, Immunity, Horses, Parasite control, Zoonoses*
orcid.org/0000-0002-0208-3861

**Dr Fabrizio Rueca, PhD**
*University of Perugia, Perugia, Italy*
***Area of expetise:*** *Lung disease, Neoplasia, Dogs*

**Dr Guillaume Sallé, PhD**
*INRA, Nouzilly, France*
***Area of expertise****: Parasitology, Nematodes, Horses, Anthelmintic resistance, Ruminants*

**Dr Francisco Miguel Sánchez Margallo, DVM, PhD**
*Jesús Usón Minimally Invasive Surgery Centre, Cáceres, Spain*
***Area of expertise:*** *Minimally invasive surgery, Laparoscopy, Endoscopy, Thoracoscopy*
orcid.org/0000-0003-2138-988X

**Dr Ken Smith, BVM&S, PhD, FHEA, FRCPath, FRCVS**
*Royal Veterinary College, London, UK*
***Area of expertise:*** *Veterinary pathology*
orcid.org/0000-0002-4861-7048

**Dr Victoria L Voith, PhD**
*Western University of Health Sciences, California, USA*
***Area of expertise:*** *Veterinary profession, Behaviour, Horses, Companion animals, Dogs*

**Prof Antonella Volta, DVM, PhD, MSc**
*University of Parma, Parma, Italy*
***Area of expertise:*** *Radiology, Ultrasound, Computed tomography*
orcid.org/0000-0002-2306-3878

**Editorial Team**

**Head of Research Operations**: Suzanne Jarvis
**Deputy Head of Research Operations**: Emma Boxer (eboxer@bvajournals.com)
**Editorial Director**: Adele Waters
**Editorial Office**
vetrecordopen@wiley.com

**Publishing Team**
**Journals Publishing Manager**: Leah Webster (lwebster@wiley.com)

### Aims and Scope

*Veterinary Record Open* is a journal dedicated to publishing specialist veterinary research across a range of topic areas, including those of a more niche or specialist nature than would be considered in *Vet Record*. Research from all disciplines of veterinary interest will be considered. It is an Open Access journal of the British Veterinary Association (BVA).

### Open Access and Copyright

All articles published by *Veterinary Record Open* are fully open access, meaning they are immediately freely available to read, download and share under the terms of the Creative Commons Attribution License, which permits use, distribution and reproduction in any medium, provided the original work is properly cited, and allows the commercial use of published articles. Copyright on any research article published by *Veterinary Record Open* is retained by the author(s). Further information about open access licenses and copyright can be found at https://bvajournals.onlinelibrary.wiley.com/hub/journal/20526113/open-access

### Disclaimer

The Publisher and Editors cannot be held responsible for errors or any consequences arising from the use of information contained in this journal; the views and opinions expressed do not necessarily reflect those of the Publisher and Editors, neither does the publication of advertisements constitute any endorsement by the Publisher and Editor of the products advertised.

Wiley Open Access articles posted to repositories or websites are without warranty from Wiley of any kind, either express or implied, including, but not limited to, warranties of merchantability, fitness for a particular purpose, or non-infringement. To the fullest extent permitted by law, Wiley disclaims all liability for any loss or damage arising out of, or in connection with, the use of or inability to use the content.

For submission instructions and all the latest information, please visit:
https://bvajournals.onlinelibrary.wiley.com/journal/20526113
